# Acknowledgment of Reviewers

**DOI:** 10.2188/jea.JE20150299

**Published:** 2015-11-05

The following scientists assisted the journal by reviewing manuscripts during the period October 1, 2014 to September 30, 2015. We gratefully acknowledge their critical evaluation and assistance in selecting articles for publication.

**Table:** 

Abe, Sarah K.	The University of Tokyo
Adachi, Hisashi	Kurume University
Akasaka, Hiroshi	Sapporo Medical University
Akiba, Suminori	Kagoshima Univeristy
Ando, Shuntaro	Tokyo Metropolitan Institute of Medical Science
Anme, Tokie	University of Tsukuba
Ansai, Toshihiro	Kyushu Dental University
Araki, Atsushi	Tokyo Metropolitan Geriatric Hospital
Arima, Hisatomi	The George Institute
Arisawa, Kokichi	The University of Tokushima
Asayama, Kei	University of Leuven
Azuma, Koichirou	Keio University
Baba, Sachiko	Osaka University
Babazono, Akira	Kyushu University
Badaloni, Chiara	Lazio Regional Health Service
Bae, Yun-Jung	Shinhan University
Bao, Wei	National Institutes of Health
Butler, Lesley	University of Pittsburgh Cancer Institute
Cable, Noriko	University College London
Cai, Lin	Fujian Medical University
Cezaretto, Adriana	University of São Paulo
Cheah, Peh-Yean	Singapore General Hospital
Chen, Pei-Chun	National Taiwan University
Chen, Renjie	Fudan University
Chihara, Izumi	University of Illinois at Chicago
Cho, Young Ae	National Cancer Center, Korea
Choi, Jeong-Hwa	Chonnam National University
Divaris, Kimon	The University of North Carolina at Chapel Hill
Doi, Yuriko	National Institute of Public Health
Eguchi, Eri	Ehime University
Ekuni, Daisuke	Okayama University
Fathallah, Fadi	UC Davis
Fujino, Yoshihisa	University of Occupational and Environmental Health, Japan
Fujiyoshi, Akira	Shiga University of Medical Science
Fukuda, Yoshiharu	Yamaguchi Unversity
Fukuhara, Masayo	Kyushu University
Fukunaga, Ichiro	Institute of Health Planning
Furuta, Michiko	Kyushu University
Gallus, Silvano	Istituto Mario Negri
Gilmour, Stuart	The University of Tokyo
Goto, Atsushi	National Center for Global Health and Medicine
Goto, Yasuyuki	Nagoya University
Grant, Eric	Radiation Effects Research Foundation
Gulis, Gabriel	University of Southern Denmark
Hagi, Yumiko	Tokai University
Hamajima, Nobuyuki	Nagoya University
Hamano, Tsuyoshi	Shimane University
Hamashima, Chisato	National Cancer Center, Japan
Hanioka, Takashi	Fukuoka Dental College
Hashimoto, Hideki	The University of Tokyo
Hashizume, Masahiro	Nagasaki University
Hata, Akira	Chiba University
Hata, Jun	Kyushu University
Hata, Kenichiro	National Research Institute for Child Health and Development
Hayashi, Kunihiko	Gunma University
Higashi, Akane	Kyoto Prefectural University
Higashiyama, Aya	National Cerebral and Cardiovascular Center
Hirao, Susumu	The Research Institute of Tuberculosis
Hirata, Takumi	Keio University
Hiratsuka, Yoshimune	Juntendo University
Hirokawa, Kumi	Baika Women’s University
Hishida, Asahi	Nagoya University
Honda, Yasushi	University of Tsukuba
Honjo, Kaori	Osaka University Global Collaboration Center
Honjo, Satoshi	Fukuoka East Medical Centre
Horiguchi, Hyogo	Kitasato University
Hoshi, Keika	Kitasato University
Hoshino, Junichi	Toranomon Hospital Kajigaya
Hosono, Akihiro	Mizuho Health Center, City of Nagoya
Hosono, Satoyo	Aichi Cancer Center Research Institute
Hsieh, Chung-Cheng	UMass Medical School
Ikeda, Ai	Juntendo University
Ikeda, Nayu	National Institute of Health & Nutrition
Imaeda, Nahomi	Nagoya Women’s University
Imai, Yutaka	Tohoku University
Imamura, Fumiaki	University of Cambridge
Inoue, Shigeru	Tokyo Medical University
Ishihara, Junko	Sagami Women’s University
Ishikawa, Hirofumi	Okayama University
Ishikawa, Shizukiyo	Jichi Medical University
Ito, Hidemi	Aichi Cancer Center Research Institute
Ito, Yuri	Osaka Medical center for Cancer and cardiovascular Disease
Iwasa, Hajime	Fukushima Medical University
Je, Youjin	Kyung Hee University
Jun, Jae Kwan	National Cancer Center, Korea
Jwa, Seung-chik	National Research Institute for Child Health and Development
Kabeya, Yusuke	Tokyo Saiseikai Central Hospital
Kachi, Yuko	Nippon Medical School
Kadota, Aya	Shiga University of Medical Science
Kakehashi, Masayuki	Hiroshima University
Kakizaki, Masako	Tohoku University
Kamo, Kenichi	Sapporo Medical University
Kanamori, Satoru	Tokyo Medical University
Kanatani, Kumiko	Kyoto University
Kanda, Hideyuki	Shimane University
Katanoda, Kota	National Cancer Center, Japan
Katayama, Akihiko	Kagawa University
Katsuya, Tomohiro	Osaka University
Kawakami, Ryoko	National Institute of Health & Nutrition
Kawamura, Takashi	Kyoto University
Kawasaki, Ryo	Yamagata University
Kayaba, Kazunori	Saitama Prefectural University
Key, Timothy	Oxford University
Khadilkar, Anuradha V	Hirabai Cowasji Jehangir Medical Research Institute, Jehangir Hospital
Khang, Young-Ho	Seoul National University
Kikuchi, Shogo	Aichi Medical University
Kim, Dae Jung	Ajou University School of Medicine
Kim, Dong Woo	National Cancer Center, Korea
Kimura, Yasutoshi	Sapporo Medical Universiy
Kita, Yoshikuni	Shiga University of Medical Science
Kitano, Naomi	Wakayama Medical University School of Medicine
Kobashi, Gen	National Institute of Radiological Sciences
Kobayashi, Nobumichi	Sapporo Medical University
Koike, Takashi	Nihon University
Kojima, Masayo	Nagoya City University
Kokubo, Yoshihiro	National Cardiovascular Center
Kondo, Naoki	The University of Tokyo
Kondo, Takaaki	Nagoya University
Koriyama, Chihaya	Kagoshima University
Kotani, Kazuhiko	Jichi Medical University
Kuchiba, Aya	National Cancer Center, Japan
Kuriyama, Shinichi	Tohoku University
Kurokawa, Naoyuki	Miyagi University of Education
Kuwahara, Erika	Toho University
Kuwahara, Keisuke	National Center for Global Health and Medicine
Lee, Joung Hee	Kyonggi University
Lee, Seungmin	Sungshin Women’s University
Lee, Yoon-Na	Korea Health Industry Development Institute
Leitzmann, Michael	University of Regensburg
Lengerich, Gene	Penn State University
Liao, Chung-Min	National Taiwan University
Lin, Yingsong	Aichi Medical Uniersity
Little, Julian	University of Ottawa
Liu, Baohua	Peking University
Liu, Jianmeng	Institute of Reproductive and Child Health, Peking University Health Science Center
Lv, Jun	Peking University
Ma, Enbo	University of Tsukuba
Marc, Isabelle	CHU de Quebec-Université Laval
Maruyama, Koutatsu	Ehime University
Matsuki, Hideaki	Tokai University
Matsumura, Yasuhiro	Bunkyo University
Matsushima, Masato	Jikei University
Matsuyama, Yusuke	Tohoku University
Matsuzaka, Masashi	Hirosaki University
Metoki, Hirohito	Tohoku University
Michikawa, Takehiro	National Institute for Environmental Studies
Minami, Ushio	University of Hyogo
Minami, Yuko	Tohoku University
Mishina, Hiroki	Kyoto University
Miyachi, Motohiko	National Institute of Health & Nutrition
Miyagawa, Naoko	Shiga University of Medical Science
Miyake, Yoshihiro	Ehime University
Mizoue, Tetsuya	International Medical Center of Japan
Mizushima, Shunsaku	Yokohama City University
Mori, Toru	Reserch Institute of Tuberculosis
Morikawa, Yuko	Kanazawa Medical University
Morisaki, Naho	National Center for Child Health and Deveopment
Murakami, Yoshitaka	Toho University
Muraki, Isao	Harvard School of Public Health
Murata, Chiyoe	Hamamatsu University
Murayama, Hiroshi	The University of Tokyo
Murayama, Hiroshi	Tokyo Metropolitan Institute of Gerontology
Murotani, Kenta	Aichi Medical University
Nagahama, Satsue	All Japan Labour Welfare Foundation
Nagai, Masato	Fukushima Medical University
Nagata, Chisato	Gifu University
Naito, Toru	Fukuoka Dental College
Nakagawa, Hiroko	Aichi Cancer Center
Nakai, Satoshi	Yokohama National University
Nakamura, Hiroyuki	Kanazawa University
Nakamura, Koshi	Hokkaido University
Nakamura, Mieko	Hamamatsu University
Nakamura, Yasuyuki	Ryukoku University
Nakata, Yoshio	University of Tsukuba
Nakatochi, Masahiro	Nagoya University Hospital
Nakaya, Naoki	Tohoku University
Nakayama, Shoji	National Institute for Environmental Studies
Ng, Chris Fook Sheng	The University of Tokyo
Ninomiya, Toshiharu	Kyushu University
Nishi, Akihiro	Yale Institute for Network Science
Nishi, Daisuke	National Disaster Medical Center
Noda, Hiroyuki	Harvard School of Public Health
Noh, Hwayoung	International Agency for Research on Cancer
Nojima, Masanori	Sapporo Medical University
Noma, Hisashi	The Institute of Statistical Mathematics
Oba, Shino	National Institute of Public Health
Odagiri, Yuko	Tokyo Medical University
Oh, Kyungwon	Korea Centers for Disease Control and Prevention
Ohira, Tetsuya	Osaka University
Ohnishi, Hirofumi	Sapporo Medical University
Ohsawa, Masaki	Iwate Medical University
Ohta, Akiko	Saitama Medical University
Ojima, Toshiyuki	Hamamatsu University
Oka, Koichiro	Waseda University
Okada, Rieko	Nagoya University
Okamoto, Kazushi	Aichi Prefectural College of Nursing and Health
Okamura, Tomonori	Keio University
Okuda, Nagako	National Institute of Health & Nutrition
Okuda, Nagako	University of Human Arts and Sciences
Onozuka, Daisuke	Kyushu University
Otsuka, Rei	National Center for Geriatrics and Gerontology
Ozasa, Kotaro	Radiation Effects Research Foundation
Qin, Li-Qiang	Soochow University
Sadakane, Atsuko	Radiation Effects Research Foundation
Saijo, Yasuaki	Asahikawa Medical College
Saika, Kumiko	Center for Cancer Control and Information Services, National Cancer Center
Sairenchi, Toshimi	Dokkyo Medical University School of Medicine
Saito, Isao	Ehime University
Saitoh, Sigeyuki	Sapporo Medical University
Sakamoto, Wataru	Okayama University
Sakurai, Masaru	Kanazawa Medical University
Sakurai, Ryota	Tokyo Metropolitan Institute of Gerontology
Sasazuki, Shizuka	National Cancer Center, Japan
Sato, Miri	University of Yamanashi
Sato, Yukihiro	Tohoku University
Satoh, Toshihiko	Kitasato University
Seki, Ayumi	Tottori University
Sheiham, Aubrey	University College London
Shibata, Yoko	Yamagata University
Shim, Jae Eun	Daejeon University
Shima, Masayuki	Hyogo College of Medicine
Shimanoe, Chisato	Saga University
Shimazaki, Yoshihiro	Aichi Gakuin University
Shin, Aesun	Seoul National University
Shinozaki, Tomohiro	The University of Tokyo
Shiraishi, Kouya	National Cancer Center Research Institute
Sho, Ri	Yamagata University
Shobugawa, Yugo	Niigata University
Sobue, Tomotaka	Osaka University
Sone, Hirohito	University of Tsukuba
Sozu, Takashi	Kyoto University
Sugiyama, Daisuke	Keio University
Sumi, Ayako	Sapporo Medical University
Sun, Xibin	Henan Cancer Hospital
Suwazono, Yasushi	Chiba University
Suzukamo, Yoshimi	Tohoku University
Suzuki, Etsuji	Okayama University
Suzuki, Kohta	University of Yamanashi
Suzuki, Yuriko	National Insitute of Mental Health, NCNP
Tabuchi, Megumi	Kwansei Gakuin University
Tabuchi, Takahiro	Osaka Medical Center for Cancer and Cardiovascular Disease
Tachimori, Hisateru	National Institute of Mental Health
Taguri, Masataka	Yokohama City University
Tajima, Kazuo	Mie University
Takahashi, Kunihiko	Nagoya University
Takashima, Naoyuki	Shiga University of Medical Science
Takebayashi, Toru	Keio University
Takegami, Misa	National Cerebral and Cardiovascular Center
Takeshita, Fumitaka	National Cancer Center Research Institute
Takeuchi, Kenji	Kyushu University
Takimoto, Hidemi	National Institute of Health & Nutrition
Tamaki, Naofumi	The University of Tokushima
Tamakoshi, Akiko	Hokkaido University
Tanabe, Naohito	Niigata University
Tanaka, Junko	Hiroshima University
Tanaka, Noriko	National Center for Global Health and Medicine
Taniguchi, Chie	Sugiyama Jogakuen University
Tanihara, Shinichi	Fukuoka University
Tanno, Kozo	Iwate Medical University
Tomita, Makoto	Tokyo Medical and Dental University
Tsakos, George	University College London
Tsuboya, Toru	Tohoku University
Tsujimoto, Takehiko	University of Tsukuba
Tsutsumi, Akizumi	Kitasato University
Turin, Tanvir	University of Calgary
Ueda, Kayo	Kyoto University
Uemura, Hirokazu	The University of Tokushima
Ukawa, Shigekazu	Hokkaido University
Urayama, Kevin	Tokyo Medical and Dental University
Usui, Tomoko	The University of Tokyo Hospital
Vogtmann, Emily	National Cancer Institute
Wada, Keiko	Gifu University
Wada, Koji	National Center for Global Health and Medicine
Wagatsuma, Yukiko	University of Tsukuba
Wakita, Takafumi	Kansai University
Wang, Haijun	Peking University
Wang, Qingsheng	Tianjin Cancer Institute and Hospital, Tianjin Medical University
Washio, Masakazu	St. Mary’s College
Watanabe, Makoto	National Cardiovascular Center
Watanabe, Shuichiro	J. F. Oberlin University
Wei, Yudan	Mercer University School of Medicine
Woo, Hae Dong	National Cancer Center, Korea
Xiang, Yong-Bing	Shanghai Cancer Institute
Yachida, Shinichi	National Cancer Center Research Institute
Yamagishi, Kazumasa	University of Tsukuba
Yamaguchi, Naohito	Tokyo Women’s Medical University
Yamamoto, Seiichiro	National Cancer Center, Japan
Yamamoto, Wari	Sapporo Medical University
Yamana, Hayato	The University of Tokyo
Yamashita, Yoshihisa	Kyushu University
Yamazaki, Shin	National Institute for Environmental Studies
Yanai, Hideki	Fukujuji Hospital
Yang, Yoon Jung	Dongduk Women’s University
Yatabe, Yasushi	Aichi Cancer Center Hospital
Yatsuya, Hiroshi	Fujita Health University
Yeh, Chih-Ching	Taipei Medical University
Yokoe, Masamichi	Japanese Red Cross Society Nagoya Daini Hospital
Yonemoto, Naohiro	National Center of Neurology and Psychiatry
Yoon, Jangho	Oregon State University
Yorifuji, Takashi	Okayama University
Yoshida, Honami	National Institute of Public Health
Yoshii, Chiharu	Wakamatsu Hospital
Yoshinaga, Jun	The University of Tokyo
Yu, Xue Qin	Cancer Council New South Wales
Zeng, Hongmei	Chinese Academy of Medical Sciences, Cancer Hospital
Zhao, Ai	Peking University
Zhao, Fang-Hui	National Cancer Center & Cancer Hospital, Chinese Academy of Medical Sciences & Peking Union Medical College
Zheng, Wei	Yamanashi University
Zou, Xiaonong	Cancer Institute, Chinese Academy of Medical Sciences

